# Deep phenotyping using foetal MRI

**DOI:** 10.1515/medgen-2026-2005

**Published:** 2026-04-16

**Authors:** Gregor Kasprian, Christian Mitter, Philipp Moser

**Affiliations:** Medical University of Vienna Division of Neuro- and Musculoskeletal Radiology, Department of Biomedical Imaging and Image-guided Therapy Währinger Gürtel 18–20 1090 Vienna Austria; Medical University of Vienna Division of Neuro- and Musculoskeletal Radiology, Department of Biomedical Imaging and Image-guided Therapy Währinger Gürtel 18–20 1090 Vienna Austria; Medical University of Vienna Division of Neuro- and Musculoskeletal Radiology, Department of Biomedical Imaging and Image-guided Therapy Währinger Gürtel 18–20 1090 Vienna Austria

**Keywords:** prenatal diagnosis, central nervous system anomalies, phenotype, neurodevelopmental disorders, fetal diseases/diagnostic imaging

## Abstract

Effectively combining prenatal ultrasound and foetal MRI maximizes the diagnostic possibilities in foetal medicine. Foetal MRI offers excellent soft tissue contrast and detailed assessment of the brain, body, and placenta. It is particularly valuable for evaluating CNS anomalies, complex syndromes, pulmonary hypoplasia, and structural abnormalities of the gastrointestinal and urogenital tracts. MRI phenotyping—performed in expert centres—enables detailed structural characterization, supporting genotype-phenotype correlations and refining risk assessment. Foetal MRI is considered safe from 18 weeks of gestation, with emerging research exploring its use earlier in pregnancy. When combined with genetic testing and expert ultrasound, foetal MRI enables a personalised, deep phenotypic evaluation. This integrated approach is essential for accurate prenatal diagnosis, risk stratification, and counselling in the context of complex congenital disorders. Rather than confirming or refuting sonographic diagnoses, MRI can be used more efficiently. Following the motto ‘the deeper you go, the more you know’, ‘MR phenotyping’ can be regarded as an important concept in future prenatal medicine.

## Introduction

In Europe, prenatal screening for congenital malformations is performed using ultrasound. Depending on national screening recommendations, first-trimester screening [1] as well as second-trimester or organ screening [2] are recommended as structured imaging procedures for the early detection of prenatal anomalies.

Over recent decades, foetal magnetic resonance imaging (MRI) has undergone significant technical and methodological advances [3]. Modern MRI techniques now allow for a detailed assessment of the developing foetus and enable a comprehensive, integrative evaluation of the brain, body, and foeto-maternal structures, including the placenta.

A major strength of foetal MRI is its excellent soft tissue contrast. This contrast is achieved by fundamentally different mechanisms compared to ultrasound. Thus foetal MRI serves as a complementary and, in selected clinical scenarios, superior diagnostic method [4]. In addition to detailed depiction of parenchymal structures, advanced imaging sequences allow for the visualisation of foetal brain connectivity via diffusion-weighted imaging [5]. Recently, even functional fetal MRI has provided insights into early human fetal brain function [6].

Modern approaches in prenatal genetics achieve significantly higher precision and success rates when they are complemented by so called “deep phenotyping”. To this end, standardized vocabulary of phenotypic abnormalities encountered in human disease – primarily from the Human Phenotype Ontology (HPO) – has been employed. Following the principle “the deeper you go, the more you know” [7], it is increasingly recognised that phenotyping can be performed at various levels of detail [8]. At specialised prenatal imaging centres that combine different modalities such as ultrasound and MRI, the concept of “*MRI phenotyping*” is implemented. *Fetal MRI phenotyping* refers to a comprehensive and detailed MRI description of structural abnormalities, normal variants, and so-called “soft markers”. These findings enhance the interpretability of genetic tests and allow a more confident genotype-phenotype correlation. The recent inclusion of prenatal structural abnormalities in the Human Phenotype Ontology (HPO) acknowledges the differences between fetal and pediatric phenotypes, underlining the importance of providing a comprehensive foetal phenotype atlas. Taking this in consideration further refined diagnostic assessment even at early gestational stages (around 20 gestational weeks) can be achieved. A thorough understanding of both, the normal development and specific pathogenic genetic anomalies and their phenotypic correlates and dynamic throughout prenatal and postnatal development is an important foundation for assessing the risk of postnatal neurocognitive, metabolic, or other comorbid conditions.

**Figure 1:**
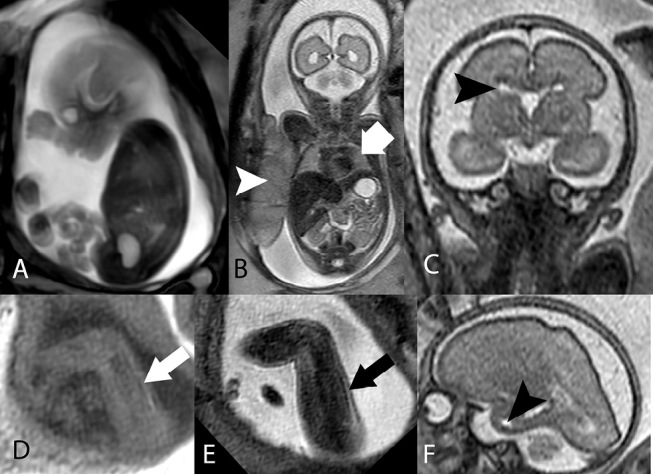
** MR Phenotyping in a case of native american myopathy (STAC3 gene mutation, OMIM: 615521):** Dynamic T2-weighted sequence of a Fetus at 28+1 GW (A), indicating bilateral talipes and camptodactyly (bent fingers); on a coronal T2-weighted sequence (B) an abnormally lobulated placenta (white arrowhead) and small lungs (white bold arrow) are visible. Coronal (C) and sagittal (F) T2-weighted images of the fetal brain show enlarged outer cerebrospinal fluid spaces as well as periventricular cysts (dark arrowheads). Sagittal T1 weighted (D) and T2-weighted sequences indicate abnormal skeletal muscle signals (T1 bright and T2 bright) indicating fatty replacement and the presence of a myopathy.

The main indications for foetal MRI include ultrasound-detected abnormalities of the foetal brain and spinal cord, assessment of complex syndromic presentations, prognostic evaluation in suspected pulmonary hypoplasia, assessment of syndromes (including congenital heart defects, and skeletal dysplasia), as well as structural anomalies of the foetal gastrointestinal and urogenital tracts.

As an example, the case of a foetal akinesia syndrome (Figure 1) is presented here. In this case, foetal MRI provided crucial phenotypic evidence for a congenital myopathy through the visualisation of signal abnormalities in foetal muscle tissue (increased T1-weighted signal suggesting fat deposition). The phenotype could be aligned with a rare case of native American myopathy [9]. The finding of periventricular cysts is novel in this case and might be associated with the concomitant finding of abnormal placental morphology as a hint towards a complex interplay between genetic and epigenetic (placental) etiologies in this case.

To date, foetal MRI has been limited to the period between the 18th week of gestation and birth, taking safety considerations into account. Long-term observations have not identified any adverse effects after fetal MRI examinations at any developmental stage of gestation, underscoring that foetal MRI – when performed using field strengths of 1.5 Tesla [10] or 3 Tesla [11] and without the use of MR contrast agents – is a safe technique [12]. This raises the possibility, from a safety perspective, of even earlier diagnostic use prior to the 18th week of pregnancy in selected cases. Ongoing MRI studies using artificial intelligence-supported image reconstruction techniques to address the problem of fetal motion at earlier gestational timepoints are currently evaluating whether this can yield additional clinically valuable information. This is particularly important as many structural foetal conditions and genetic diagnoses are now identified well before the 18th week of gestation [13]. Establishing early structural genotype-phenotype correlations during early pregnancy could therefore make a significant contribution to prenatal medicine. Currently, the main challenges in achieving this goal are not primarily technological but lie in the practical and educational limitations of the method. Only a few centers worldwide have developed a sufficiently deep expertise that allows the implementation of the method in routine settings. To reduce the expertise-dependent interobserver variability, broader exposure and further integration into (neuro)radiological and fetomaternal fellowship programs will be required[14]. This will demand an open and integrated approach that transcends disciplinary boundaries in prenatal imaging in favor of a more patient-centered care.

The following sections provide an overview of the main applications of foetal neuroimaging, as well as future perspectives and the role of the method in combination with genotyping.

### Foetal MRI of the central nervous system (CNS)

The primary application of foetal MRI lies in the evaluation of the foetal CNS. It serves as a crucial complement to foetal ultrasound and neurosonography. Current guidelines recommend the use of foetal MRI either in addition to transabdominal ultrasound or as a complement to, or even replacement for, foetal neurosonography [3]. In the complementary approach practised, for instance, at the Vienna centre, foetal neuro-MRI can compensate for the physical and technical limitations of ultrasound, thereby significantly enhancing diagnostic accuracy. For example, foetal MRI using susceptibility-weighted sequences (echo planar sequences, T2* sequences) shows high sensitivity in detecting residual haemorrhages, whereas prenatal ultrasound is particularly strong in identifying calcifications and visualising the choroid plexus. The additive value of both methods is usually incompletely objectified using quantitative scientific approaches, as differences in image quality, foetal MRI expertise, and diagnostic goals (diagnosis versus prognostic/personalized fetal imaging) must also be considered in comparative studies of ultrasound and MRI [4, 15]. Even if the final diagnosis of – for instance – “agenesis of the corpus callosum” remains semantically unchanged following MRI assessment, a more detailed, MRI-based phenotyping can depict the spectrum of structural abnormalities more precisely – which in turn facilitates diagnostic classification and counselling by paediatric neurologists [16].

**Figure 2:**
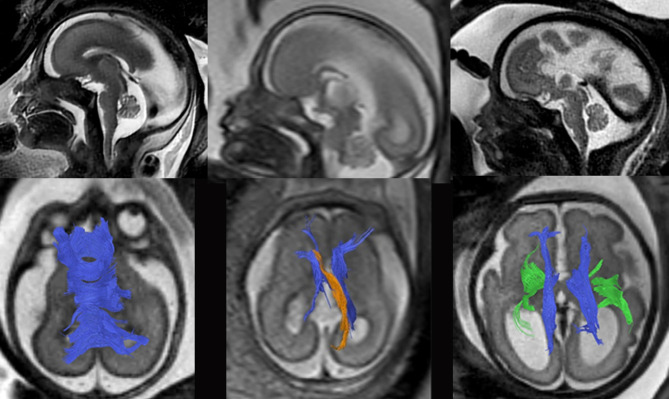
**Variations of midline morphology in three cases with proven** ***ARID1B* mutations:** Left row: structurally normal corpus callosum on median sagittal T2-weighted sequences (upper row) as well as on 3D tractography at 24GW. Middle row: partial callosal agenesis at 24GW with the presence of a sigmoid bundle (orange, lower row). Right row: 29GW week fetus with *ARID1B* mutation and complete callosal agenesis and Probst bundles (blue, lower row).

A short foetal MRI scan of approximately 10 to 15 minutes is sufficient to enable comprehensive, artefact-free phenotypic evaluation of all brain structures (a complete foetal MRI scan can take up to 45 minutes). These examinations are performed by experienced radiographers and specialist physicians with theoretical and practical expertise in foetal MRI. For image interpretation knowledge on the normal course of fetal brain development as well as proficiency in the field of fetal MR image acquisition is essential [17]. Comparison to normal age matched reference cases – recently accessible through fetal brain atlases [18] – is especially helpful to unexperienced examiners. Structured educational courses and guided exposure to actively performing and guiding fetal MRI examinations are being established to guarantee adhesion to reporting standards in fetal neuroimaging [14].

Fetal Neuro-MRI examinations allow for the detailed qualitative – and in some cases quantitative – visualisation of key foetal brain structures, including the corpus callosum, hippocampus, all brain regions of cortical folding considering normal cortical development according to gestational age, basal ganglia [19], thalami, the foetal brainstem [20] and its substructures, the cerebellum including the vermis cerebelli [21], as well as the olfactory bulb, optic nerves and pituitary gland. With appropriate expertise, these structures should be visualised in 100 % of all MRI examinations between 18 GW and birth. Furthermore, T2-weighted sequences [22] and recently adapted Fluid Attenuated Inversion Recovery (FLAIR) sequences [23] allow even more precise depiction of the individual layers of the foetal brain parenchyma.

This sets foetal MRI apart from transabdominal ultrasound and neurosonography, in which the visualisation rates of these fine foetal brain substructures are lower depending on operator expertise [24]. In addition, the position of the foetal head (breech or cephalic) is of little relevance for visualising the aforementioned structures using conventional T2-weighted MRI sequences. In contrast, foetal neurosonography requires skill in manual techniques such as internal rotation to move the foetus into a cephalic position close to the transvaginal probe. Even after such manoeuvres, completely artefact-free depiction of cortical folding and the posterior fossa is not guaranteed, particularly in later stages of pregnancy (after 30 weeks of gestation), due to bone shadowing artefacts [25, 26]. However, a profound assessment of complex cerebral malformations requires the evaluation of all brain structures clearly and completely, in order to enable the most accurate prognostic counseling in conjunction with genetic findings. The most significant current limitation of foetal MRI remains its limited availability, which depends heavily on the expertise of specialised examiners and the access to global high quality training opportunities [3].

An illustrative example is the sonographic detection of fetuses with agenesis of the corpus callosum. The sonographic detection of an absent corpus callosum should be achieved by any standard prenatal ultrasound examination at around 20 GW and earlier. After sonographic detection, many centers request fetal MRI examinations – usually at around 30GW, in expert centers also earlier – immediately after sonographic diagnosis. Thus, agenesis of the corpus callosum is one of the most common indications for a foetal MRI scan. Despite the most careful ultrasound and neuroradiological MRI assessment, it is often difficult to predict the neurocognitive prognosis in foetuses and children with corpus callosum agenesis.

This is partly due to the fact that complex syndromes involving significant functional impairments can be hidden behind only mildly abnormal structural findings. One notable example is Coffin-Siris syndrome, which is one of the most frequent genetic causes of complete or partial agenesis of the corpus callosum [27]. Conversely, incomplete phenotyping may lead to an underestimation of the potential risk profile and thus an overly optimistic prognosis. Figure 2 exemplifies the phenotypic variability of three different cases with *ARID1B* mutations using in utero Diffusion tensor imaging (DTI) and white matter visualization in 3D using tractography. Despite identical genetic diagnosis, tractography shows phenotypic variants with typical changes in complete or partial callosal agenesis with Probst bundles and/or Sigmoid bundles or even a normal callosal appearance.

Here, foetal MRI can make a decisive contribution: high-resolution depiction of the smallest anatomical substructures enables the identification of subtle soft markers and associated structural changes, whose cumulative presence increases the likelihood of an underlying genetic condition and thus the probability of more serious developmental neurological impairments [28].

**Figure 3:**
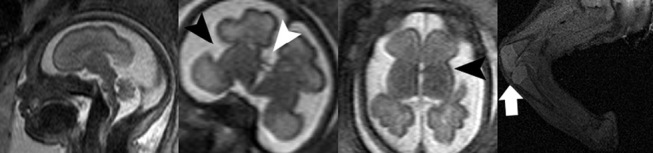
** Fetal MRI in a case of genitopatellar syndrome at 32GW:** note the delayed opercularization (black arrowhead, usually the insula should be covered by the adjacent frontal and temporal lobes), the absence of the corpus callosum (white arrowhead) as well as the simplified gyral pattern. The pathognomonic absence of the patella can be seen by fetal MRI as well, however is frequently missed – due to subtlety of this finding. In this case absence of the patella was diagnosed postnatally (white arrow).

Figure 3 shows MRI images of a foetus with complete agenesis of the corpus callosum along with additional structural abnormalities, including significantly delayed opercularisation, simplified gyral pattern and small dimensions of the forebrain. The characteristic absence of the patella could only be diagnosed retrospectively in knowledge of the postnatal imaging findings. In this case, genetic findings were consistent with genitopatellar syndrome. This case is representative for many congenital neurologic abnormalities, showing a variety of phenotypic features, which – evaluated in isolation – are unspecific for a certain syndrome, however taken together, increase the likelihood of a specific positive genetic finding and thus can guide phenotype specific deeper assessment of high throughput exome or genome data and ultimately increase the genetic diagnostic yield [16, 29]. Of the more than 50 known genetic disorders associated with corpus callosum agenesis, none display a specific or consistently expected foetal neuroradiological pattern. Many of these syndromes show considerable phenotypic variability. Only through detailed phenotypic characterisation using foetal MRI – which can capture even subtle changes such as hippocampal positioning or brain parenchyma organisation – is it possible to achieve the most holistic prognostic classification, risk assessment, and correlation with genetic findings.

**Figure 4:**
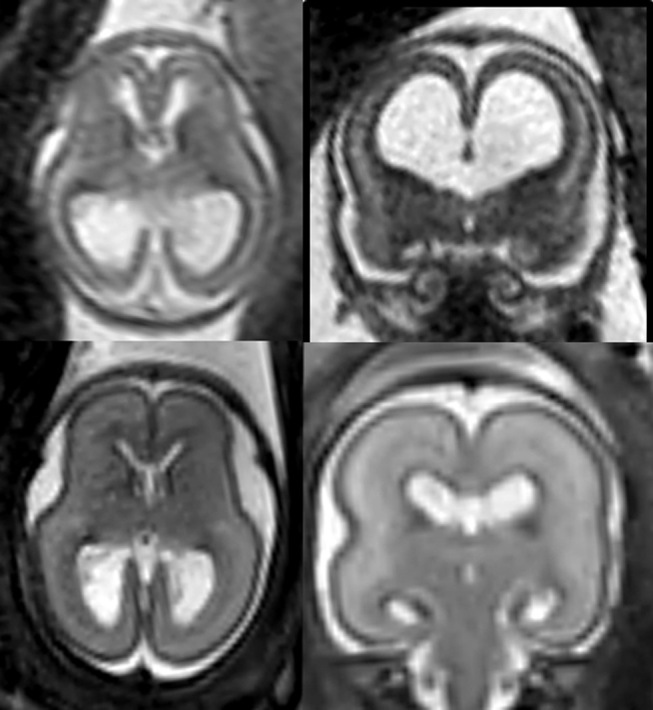
** Fetal MRI in a case of Witteveen-Kolk Syndrome (OMIM: 613406)** at 18+2 (upper row) and 23+4GW (lower row). Initially severe ventriculomegaly was found at sonography and confirmed by MRI at 18+2GW. While ventricular width was normalizing at 23+4GW, there were several MR softmarkers indicating the presence of a more complex phenotype: Delayed opercularization and verticalization of hippocampi, thalamic fusion and fusion of the fornices.

A similar situation arises in the assessment of cases with enlarged lateral ventricles of varying severity – from mild to moderate to severe ventriculomegaly (see Figure 4). Even mild or progressive ventriculomegaly may, in some cases, be associated with significant developmental delays [30]. When performed by expert fetal MR imaging teams, MRI is recommended and its diagnostic yield is dependent on the quality of the preceding sonographic examination [30].

Figure 4 illustrates the foetal MRI findings in a foetus with Witteveen-Kolk syndrome. In this case, despite otherwise normal cortical folding, multiple subtle abnormalities are present, including delayed opercularization, fusion of thalami and fornices and delayed hippocampal rotation. While these individual findings may also occur within the spectrum of normal variants, the combination of these structural abnormalities, together with ventriculomegaly, is associated with a significantly increased likelihood of genetic or developmental neurological impairments. Witteveen-Kolk syndrome is typically associated with moderate to severe neurocognitive developmental impairments [31]. Even though there was a reduction of ventricular width at follow-up, MRI indicated several soft markers. While these imaging features were non-specific, their comprehensive assessment via foetal MRI refined the risk profile and ultimately allowed for a clear differentiation from uncomplicated mild ventriculomegaly.

These cases presented here are exemplaric for some genetic disorders and their potential fetal phenotype. Currently, we are only at the beginning of understanding the phenotypic spectrum of monogenetic disorders. As more prenatal imaging data is emerging, our knowledge of genotype-phenotype interactions at prenatal stages is deepening. In some cases – such as *ARID1B* (Figure 2) or *DCC* mutations [32] in callosal agenesis a broad prenatal phenotypic spectrum is evolving. In other cases certain imaging patterns – such as cavitation of ganglionic eminences paired with callosal agenesis [33] in pyruvate dehydrogenase deficiency – are found as more specific markers for certain entities. Future efforts in optimizing phenotypic biomarker descriptions in HPO terms and combining certain imaging patterns – best described as “phenomes” – will certainly help in optimizing the precision of counseling and phenotype prediction.

### Foetal MRI in complex syndromic malformations

Complex malformation syndromes involving multiple organ systems require the closest possible correlation between genetic and structural findings. In this context, foetal MRI offers particular strengths, as it enables high-precision, three-dimensional, and volumetric assessment of various foetal organ systems.

A key application lies in the evaluation of foetal lung volumes, which are of prognostic importance especially in foetuses with congenital diaphragmatic hernia [34] or oligohydramnios [35] – for example, due to premature rupture of membranes or renal malformations.

Foetal MRI also provides valuable information regarding the gastrointestinal tract: the high contrast of meconium, which appears hyperintense on T1-weighted sequences, allows for precise differential diagnosis of the level of intestinal stenosis and improved detection of high anorectal atresia.

Diffusion-weighted sequences enable detailed depiction of foetal renal parenchyma, which, due to its micro architecture, appears highly isotropic (bright on diffusion-weighted sequences). This allows for conclusive assessment of residual functional parenchyma, particularly in cases of multicystic kidney disease [36].

Foetal MRI can also provide important additional information in complex metabolic disorders such as mitochondrial diseases. In addition to structural cerebral abnormalities and malformations, metabolic changes can be suspected based on characteristic signal alterations in the liver [37].

Furthermore, differentiation between fetal infections (TORCH) and pseudo-TORCH syndromes can be aided by the combination of structural and signal-based findings, providing crucial information for further genetic or infectious disease evaluation. In addition to the reliable detection of hepatosplenomegaly, MR imaging findings of the placenta play a central role.

Placental abnormalities such as infarctions, haemorrhages, or premature maturation patterns can serve as indicators of previous or ongoing chorioamnionitis, contributing to the differentiation between syndromic, acquired, and genetic conditions [38].

## Conclusion

Fetal MRI is a valuable adjunct to prenatal ultrasound. In order to achieve deep phenotypic characterizations of fetal disorders, a complete, personalized imaging evaluation as well as deep knowledge of normal fetal imaging according to gestational age are both essential. By combining excellent prenatal ultrasound with expert fetal MRI, a holistic evaluation of the fetus and the placenta can be achieved. As most congenital disorders are not only biologically complex but also vary widely in severity and future functional phenotype, all levels of characterization (genomes, phenomes – and even connectomes) need to be included in their evaluation. As prenatal imaging data gets more detailed and complex, the process of integrating prenatal phenotyping through multimodal imaging and modern genomic analysis has just begun. Promoting an integrative multi-level understanding of prenatal disorders is crucial to promote personalized medicine at this early stage of life and ultimately to treat fetuses as patients.

## References

[1] Boyd P, Devigan C, Khoshnood B, Loane M, Garne E, Dolk H (2008). Survey of prenatal screening policies in Europe for structural malformations and chromosome anomalies, and their impact on detection and termination rates for neural tube defects and Down’s syndrome. BJOG: An International Journal of Obstetrics &amp; Gynaecology.

[2] Salomon LJ, Alfirevic Z, Berghella V (2022). ISUOG Practice Guidelines (updated): performance of the routine mid‐trimester fetal ultrasound scan. Ultrasound in Obstetrics &amp; Gynecology.

[3] Prayer D, Malinger G, De Catte L (2023). ISUOG Practice Guidelines (updated): performance of fetal magnetic resonance imaging. Ultrasound in Obstetrics &amp; Gynecology.

[4] Griffiths PD, Bradburn M, Campbell MJ (2017). Use of MRI in the diagnosis of fetal brain abnormalities in utero (MERIDIAN): a multicentre, prospective cohort study. The Lancet.

[5] Kasprian G, Brugger PC, Schopf V (2013). Assessing prenatal white matter connectivity in commissural agenesis. Brain.

[6] Thomason ME, Palopoli AC, Jariwala NN (2021). Miswiring the brain: Human prenatal Δ9-tetrahydrocannabinol use associated with altered fetal hippocampal brain network connectivity. Developmental Cognitive Neuroscience.

[7] Delude CM (2015). Deep phenotyping: The details of disease. Nature.

[8] Robinson PN (2012). Deep phenotyping for precision medicine. Human Mutation.

[9] Grzybowski M, Schänzer A, Pepler A, Heller C, Neubauer BA, Hahn A (2017). Novel STAC3 Mutations in the First Non-Amerindian Patient with Native American Myopathy. Neuropediatrics.

[10] Strizek B, Jani JC, Mucyo E (2015). Safety of MR Imaging at 1.5 T in Fetuses: A Retrospective Case-Control Study of Birth Weights and the Effects of Acoustic Noise. Radiology.

[11] Barrera CA, Francavilla ML, Serai SD (2020). Specific Absorption Rate and Specific Energy Dose: Comparison of 1.5-T versus 3.0-T Fetal MRI. Radiology.

[12] Ray JG, Vermeulen MJ, Bharatha A, Montanera WJ, Park AL (2016). Association Between MRI Exposure During Pregnancy and Fetal and Childhood Outcomes. JAMA.

[13] Von Kaisenberg C, Kozlowski P, Kagan K-O (2024). Firsttrimester Diagnosis and Therapy @ 11–13+6 Weeks of Gestation – Part 1. Geburtshilfe und Frauenheilkunde.

[14] Prayer D, Malinger G, De Catte L (2023). ISUOG Practice Guidelines (updated): performance of fetal magnetic resonance imaging. Ultrasound Obstet Gynecol.

[15] Malinger G, Ben‐Sira L, Lev D, Ben‐Aroya Z, Kidron D, Lerman‐Sagie T (2004). Fetal brain imaging: a comparison between magnetic resonance imaging and dedicated neurosonography. Ultrasound in Obstetrics &amp; Gynecology.

[16] Glatter S, Kasprian G, Bettelheim D (2021). Beyond Isolated and Associated: A Novel Fetal MR Imaging–Based Scoring System Helps in the Prenatal Prognostication of Callosal Agenesis. American Journal of Neuroradiology.

[17] Prayer D, Kasprian G, Krampl E (2006). MRI of normal fetal brain development. European journal of radiology.

[18] Gholipour A, Rollins CK, Velasco-Annis C (2017). A normative spatiotemporal MRI atlas of the fetal brain for automatic segmentation and analysis of early brain growth. Sci Rep.

[19] Stuempflen M, Taymourtash A, Kienast P (2023). The ganglionic eminence: volumetric assessment of transient brain structure utilizing fetal magnetic resonance imaging. Ultrasound in Obstetrics &amp; Gynecology.

[20] Dovjak GO, Schmidbauer V, Brugger PC (2020). Normal human brainstem development in vivo: a quantitative fetal MRI study. Ultrasound in Obstetrics & Gynecology.

[21] Dovjak GO, Brugger PC, Gruber GM (2018). Prenatal assessment of cerebellar vermian lobulation: fetal MRI with 3-Tesla postmortem validation. Ultrasound in obstetrics & gynecology : the official journal of the International Society of Ultrasound in Obstetrics and Gynecology.

[22] Pogledic I, Schwartz E, Mitter C (2020). The Subplate Layers: The Superficial and Deep Subplate Can be Discriminated on 3 Tesla Human Fetal Postmortem MRI. Cerebral Cortex.

[23] Diogo MC, Prayer D, Gruber GM (2019). Echo-planar FLAIR Sequence Improves Subplate Visualization in Fetal MRI of the Brain. Radiology.

[24] Fernando S, Lavender I, Coombs P (2025). Visualization of the Fetal Corpus Callosum in Routine Second Trimester Screening Ultrasound Examinations. Journal of Ultrasound in Medicine.

[25] Codaccioni C, Arthuis C, Deloison B (2025). Offline ultrasound MRI fusion imaging for assessment of normal fetal brain development. Ultrasound in Obstetrics &amp; Gynecology.

[26] Pertl B, Eder S, Stern C, Verheyen S (2019). The Fetal Posterior Fossa on Prenatal Ultrasound Imaging: Normal Longitudinal Development and Posterior Fossa Anomalies. Ultraschall in der Medizin – European Journal of Ultrasound.

[27] Martins-Costa C, Pham VA, Wiegers A (2023). ARID1B controls transcriptional programs of axon projection in the human corpus callosum. Cold Spring Harbor Laboratory.

[28] Diogo MC, Glatter S, Prayer D (2021). Improved neurodevelopmental prognostication in isolated corpus callosal agenesis: fetal magnetic resonance imaging‐based scoring system. Ultrasound in Obstetrics & Gynecology.

[29] Heide S, Spentchian M, Valence S (2020). Prenatal exome sequencing in 65 fetuses with abnormality of the corpus callosum: contribution to further diagnostic delineation. Genetics in Medicine.

[30] Fox NS, Monteagudo A, Kuller JA, Craigo S, Norton ME (2018). Mild fetal ventriculomegaly: diagnosis, evaluation, and management. American Journal of Obstetrics and Gynecology.

[31] Van Dongen LCM, Wingbermühle E, Dingemans AJM (2020). Behavior and cognitive functioning in Witteveen–Kolk syndrome. American Journal of Medical Genetics Part A.

[32] Marsh AP, Heron D, Edwards TJ (2017). Mutations in DCC cause isolated agenesis of the corpus callosum with incomplete penetrance. Nature genetics.

[33] Fortin O, Christoffel K, Shoaib AB (2024). Fetal Brain MRI Abnormalities in Pyruvate Dehydrogenase Complex Deficiency. Neurology.

[34] Da-Costa-Santos J, Bennini JR (2022). Imaging Assessment of Prognostic Parameters in Cases of Isolated Congenital Diaphragmatic Hernia: Integrative Review. Revista Brasileira de Ginecologia e Obstetrícia / RBGO Gynecology and Obstetrics.

[35] Messerschmidt A, Pataraia A, Helmer H (2011). Fetal MRI for prediction of neonatal mortality following preterm premature rupture of the fetal membranes. Pediatric Radiology.

[36] Gaunt T, Sokolska M (2023). Physiological assessment of the fetal body using MRI: current uses and potential directions. Br J Radiol.

[37] Schwarz M, Schmidbauer VU, Malik J (2024). Intrauterine blood transfusion causes dose- and time-dependent signal alterations in the liver and the spleen on fetal magnetic resonance imaging. Eur Radiol.

[38] Messerschmidt A, Baschat A, Linduska N (2011). Magnetic resonance imaging of the placenta identifies placental vascular abnormalities independently of Doppler ultrasound. Ultrasound Obstet Gynecol.

